# Bone Surface Mapping Method

**DOI:** 10.1371/journal.pone.0032926

**Published:** 2012-03-08

**Authors:** Yifang Fan, Yubo Fan, Zhiyu Li, Changsheng Lv, Bo Zhang

**Affiliations:** 1 Center for Scientific Research, Guangzhou Institute of Physical Education, Guangzhou, People's Republic of China; 2 Key Laboratory for Biomechanics and Mechanobiology of Ministry of Education, School of Biological Science and Medical Engineering, Beihang University, Beijing, People's Republic of China; 3 College of Foreign Studies, Jinan University, Guangzhou, People's Republic of China; Institution of Automation, CAS, China

## Abstract

Bone shape is an important factor to determine the bone's structural function. For the asymmetrically shaped and anisotropically distributed bone *in vivo*, a surface mapping method is proposed on the bases of its geometric transformation invariance and its uniqueness of the principal axes of inertia. Using spiral CT scanning, we can make precise measurements to bone *in vivo*. The coordinate transformations lead to the principal axes of inertia, with which the prime meridian and the contour can be set. Methods such as tomographic reconstruction and boundary development are employed so that the surface of bone *in vivo* can be mapped. Experimental results show that the surface mapping method can reflect the shape features and help study the surface changes of bone *in vivo*. This method can be applied to research into the surface characteristics and changes of organ, tissue or cell whenever its digitalized surface is obtained.

## Introduction

The shape of bone is the result of adaptation to mechanical environment [1], [2], [3], [4] and in return, the shape affects the structural function of bone [5], [6]. A map is a figure of a spherical surface drawn on a plane surface according to mathematical rules [7], [8]. The drawing method is called development. For example, the boundary of a circle can be developed as a straight line. When the bone is simplified as a set of finite tomographic images, the bone's surface is a set of tomographic boundaries. When the closed tomographic boundary is developed according to mathematical rules, the bone's surface map is thus formed by a set of developed lines. A bone's surface map is a way to reveal the bone's shape. The bone surface mapping, therefore, is used as an approach to study the adaptability of bone and its structural function.

Mapping and flattening methods have been widely used in medical research [9], [10], [11], [12], [13]. They are both concerned with the development methods (i.e. drawing methods according to mathematical rules). The flattening method develops the 3-dimensional (3D) object to a 2-dimensional (2D) one while the mapping method plays an essential role in interpreting the surface structure of an object [14]. The advancements and improvements of 3D imaging of bone *in vivo* [15], [16], [17] have brought better methods to digitalize the surface of bone, but the mapping methods have not been systematically explored with satisfactory results. Specifically, how to establish a unique coordinate system of bone *in vivo* has not been introduced, but to set up a unique coordinate system is a prerequisite to draw the bone's surface map.

In the spherical coordinate system, the position of earth surface point is determined by the longitude, latitude and altitude [18]. The prime meridian (0° longitude) and the equator line (0° latitude) are the origin of a rectangular (or Cartesian) coordinate system [19]. Apart from the size difference, the bone and the earth are both a 3D geometry. In order to draw a bone's surface map, these three quantities need to be defined.

The altitude of the earth refers to the vertical distance of something above a reference point that usually refers to the sea level (i.e. the average distance from the sea surface [8]). As to the bone's altitude, it can be defined when the sea level is expressed as mean distance of the bone's surface points relative to the center of mass (COM). The prime meridian and equator line of the earth are artificial definitions [8], which bring difficulty to the definition of bone's prime meridian and equator line.

The magnitude of an object's moment of inertia is related not only to the object's mass but also to its mass distribution. It is a tensor whose form is similar to a 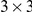 matrix [20]. The three diagonal elements of the tensor are the three axis moments of inertia of an object, respectively. The sum of these three axis moments of inertia doubles the moment of inertia of that object [21]. To an asymmetrical object, the magnitude of the three axis moments of inertia is determined by its body coordinate while their sum remains unvaried. Using the characteristics of an object's axis moment inertia, for an object like bone, when the origin of the rectangular coordinate system set by the measurement equipment is moved to the bone's COM, the bone's body coordinate system is formed. When the coordinate transformation, i.e. coordinate system rotates around bone's origin, is conducted to the bone's body coordinate system, a coordinate system with the products of inertia (i.e. the off-diagonal elements of a tensor) to be zero can be identified [20]. The axis of the new coordinate system is the bone's principal axis of inertia (PAI).

When the bone's PAI is applied to determine the prime meridian and equator line of a 3D model, we must make sure that the bone's shape will not be affected by the coordinate transformation. The scalar remains invariant under coordinate transformation [22]. The bone's surface can be presented by the finite point clouds. The point cloud position is a scalar, which means that the bone's shape will not be affected by the coordinate transformation [23]. The pairwise orthogonal PAIs can form three bone cross sections. The boundaries of the two sections vertical to PAI (the magnitude of principal moments of inertia is minimal or maximal) are to represent the prime meridian and equator line, respectively. Then, the bone's prime meridian and the equator line can be determined by the bone's PAIs.

This paper examines and verifies the uniqueness of PAIs of the asymmetrically shaped and anisotropically structured bone *in vivo*. The experimental results suggest that when PAI is used to set the prime meridian and when the average radius of the tomographic boundary (relative to PAI) is applied to set the contour, an approach to map the bone surface is practical. This bone surface mapping method provides an alternate method to study the bone's morphology. It can also be employed to study the surface characteristics and the changes of organ, tissue or cell.

## Materials and Methods

### Ethic Statement

The study received approval from the Ethical Committee of Guangzhou Institute of Physical Education. The subjects provided fully informed consent to participate in this study by signing a written consent form.

### Equipment

The test equipment was Brilliance 64-slice Scanner by Philips, Netherlands, provided by Image Processing Center of Zhujiang Hospital. Scan settings were: frame bone tissue; power: 120 kv; pixel size: 0.50 mm; layer distance: 0.50 mm. The scanning was conducted along both feet transect, from top to bottom.

### Software

The 3D model was constructed by *Mimics* (Version 10) and *Geomagic Studio*, and the statistical analysis was performed by *SPSS* (Version 12) (provided by the Key Laboratory of Biomechanics and Mechanobiology of Ministry of Education).

### Participants

Altogether, we collected bone data of 16 athletes - 8 volleyball players (with average height, weight and age of 183.94±3.90 cm, 69.80±5.20 kg and 21.88±0.99 yrs, respectively) and 8 wrestlers (with average height, weight and age of 168.00±5.68 cm, 65.52±5.16 kg and 21.00±2.78 yrs, respectively).

The subjects were male volleyball players from our institute and male wrestlers from Provincial Sports School. It was confirmed before the test that every subject had been trained as a professional athlete for more than five years. Before the test, each subject's medical history was inquired and all the subjects were *x*-rayed to exclude subjects with diseases such as foot pathological change, deformity or injury to make sure that their physical conditions meet the requirements of the test.

### Standardized Coordinate System of Bone

An isotropic scanning separates the bone into a collection of volume element (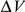). After the scanning, the coordinates of volume element become known. Then the COM can be obtained after calculation. Let the coordinates of volume element relative to COM be 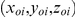 (where *o* is located in COM), and then the magnitude of the principal moments of inertia is represented by:
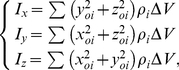
(1)where 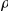 is the density of volume element, 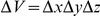, where 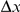, 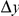 and 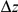 are the horizontal, vertical pixel sizes and the layer distance of CT images, respectively.

Let the angles made with the *x*, *y*, *z* axes be 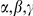. The following equations are set up:
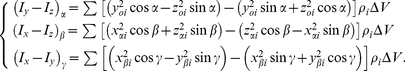
(2)Differentiate Eq. (2), and let
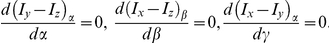
Then
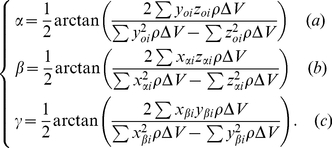
(3)Eq. (3) shows that if and only if 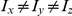 will Eqs. (3a)–(3c) have a set of solutions. In Eq. (3), 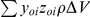, 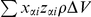 and 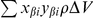 are products of inertia of inertia tensor. Within the range of 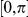, according to Eq. (3), limited rotations find a set of body axes for which the products of inertia vanish.

### Reconstruction

When the result of Eq. (3) is replaced for that in Eq. (2) accordingly, a set of bone body axes is positioned on bone's PAI. Since the tomographic images are numerical, the transformation leads to changes of the original tomographic images such as the tomographic area and boundary. It is necessary, then, to reconstruct a tomographic image after transformation, which can be performed by the following equation:
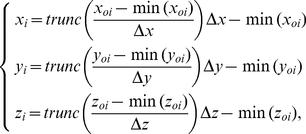
(4)where 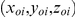 stands for the position of volume element after transformation, 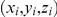 for that of the reconstructed tomogram and *trunc()* for a function that truncates a number to an integer by removing the fractional part of the number. To keep the CT images isotropic, Eq. (4) is satisfied only by 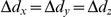, and pixel size and layer distance are kept the same of (or a little more than) those of the original image.

### Boundary detection

CT scanning separates the bone surface into a collection of the tomographic image boundaries. The mapping of the bone surface has become an issue to develop the tomographic boundary. When performing a CT scanning, the equipment is reset, i.e. the gray value of the air is set as zero. The scanned tomographic images of bone are restructured by Eq. (4), and their tomographic boundaries are drawn by the following equation:
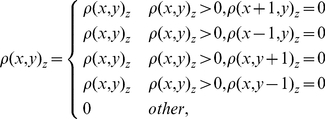
(5)where z is the sequence number of tomographic images, 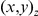 the position of volume element and 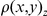 the density of volume element.

### Cutting line

When mapping the bone surface, the bone is cut – from a cylindrical surface to a rectangle, or a rhombus. However the cylindrical surface is cut, its ultimate area would be the same. But there is only one in shape of rectangle.

How to cut the bone into only one form? Two PAIs are in a plane perpendicular to the minimal or maximal PAI of bone. On this plane, the bone surface boundary is defined as the prime meridian, which is used as the surface cutting line to develop the bone surface. The following equation makes it happen:
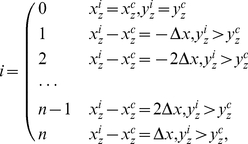
(6)where 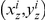 is the position of the volume element at the tomographic boundary, 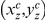 the COM relative to the inertia reference frame and *i* the sequence number of volume element at the boundary after being cut.

### Contour

Eq. (6) sequences the volume elements at the tomographic boundary which has been cut. The average radius of the tomographic image perpendicular to PAI is defined as the sea level, which is used as a datum line so that the tomographic boundary can be developed by the following equation:
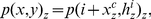
(7)where *z* shares the same definition of that in Eq. (5), 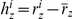, 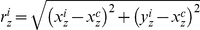 and 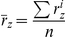, 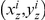 and 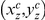 have the same definitions with those in Eq. (6).

### Flattening

Eqs. (4)–(7) develop the closed surface into an open 3D curved one. The 3D curved map of the bone surface can be further developed into a 2-D plane. We translate the bone surface into a plane by the following equation:
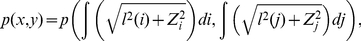
(8)where 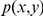 presents the position of volume element in a plane whose surface map has been flattened. 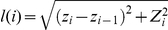, 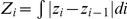, 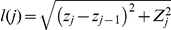 and 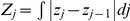, where *z* stands for the value of contour on 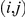 in bone surface mapping.

Eqs. (4)–(8) suggest that the mapping of the bone surface actually serves as a simulation of the bone surface structure. It is a space model of an image symbol to represent the bone surface. It remains consistent with the real body of the bone surface structure.

## Results and Discussion

The inertia tensor suggests that we can find a set of body axes where products of inertia will be zero at the same time [20], [24], [25]. Three principal moments of inertia are formed relative to PAI. The magnitudes of these three principal moments of inertia come out with only three results: (i) all are equal; (ii) two out of three are equal and (iii) all are different. When the object is homogeneous, in the first case, it is a sphere; in the second, an ellipsoid, a cube, a cylinder or a rectangular.

In the first case, there are numerous coordinate systems set upon PAIs, while in the second case, the orientation of one principal axis is determined, but not the other two. Therefore, in the first and second case, there is not a corresponding relation between PAIs and the shape. In the third case, however, the direction cosines of the bone's PAI relative to inertia reference frame are unique, suggesting a one-to-one corresponding relation between PAIs and shape.

**Figure 1:**
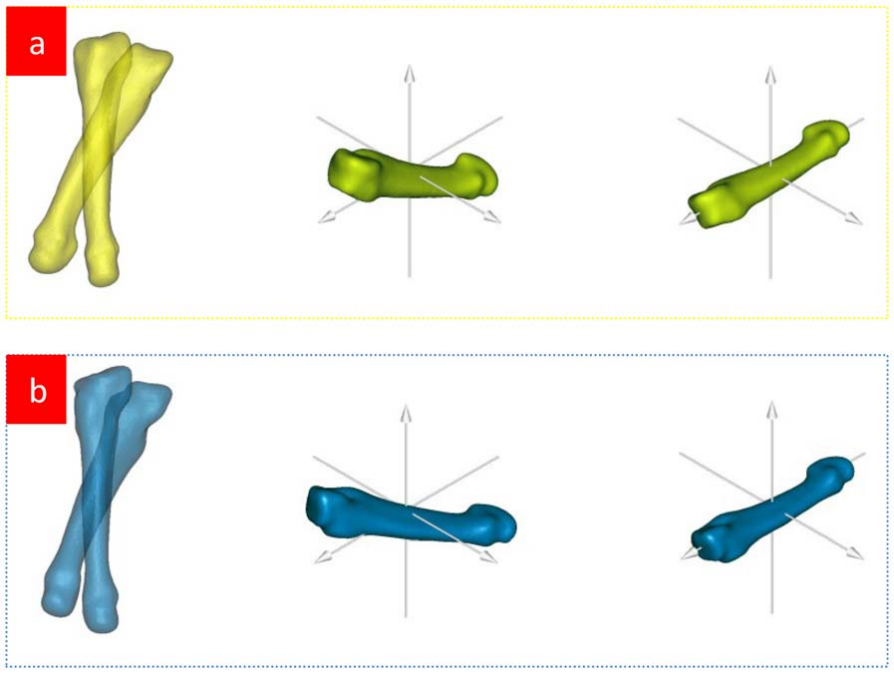
SCS of the fourth metatarsal. Fig. 1a SCS of the fourth metatarsal of the wrestler. Fig. 1b SCS of the fourth metatarsal of the volleyball player. The application of *Mimics* software leads to the 3D model of the fourth metatarsal. The 3D model is then reconstructed and saved as text file by “point cloud” process (the menu in the *Mimics*). Applying Eq. (3), SCS is conducted and then *Geomagic Studio* is used to wrap or smooth shade the processed point cloud data. Next, save the 3D model as a STL (standard template library) file. Last, input the standardized 3D model of the fourth metatarsal into *Mimics*. The left figure is a comparison of bone before and after using SCS, the middle figure is the posture of bone in body coordinate system before using SCS while the right one is the posture of bone in body coordinate system after using SCS. When the coordinate of the equipment is translated to the bone's COM, two different postures of the two subjects' fourth metatarsal can be spotted. But after using SCS, the bone's body coordinate axes are translated into the bone's PAIs, where the postures can be unified in the coordinate system.

The magnitude of the bone's three principal moments of inertia is determined by its mass and mass distribution. As to the bone, whose shape is asymmetrical and its distribution non-homogeneous [26], [27], [28], will the third case be applied? This needs experimental verification. Using *Mimics* software, a reconstruction to the CT image is conducted and a 3D model of the athlete's left foot bone is built. The fourth metatarsal of the athlete is picked. Now, look at the result from the standardized coordinate system (SCS) of bone, i.e. body coordinate system is transformed into PAIs. See Figure 1.

This shows, when 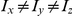, a set of body axes whose coordinate origins are located at the COM of bone are set up. After limited rotations, PAIs can be spotted. The direction cosines of bone's PAI relative to the inertia reference frame characterization are unique (i.e. the third case), which means that the body axes set upon PAIs not only depict the position and posture of bone, but also verify the bone surface shape and its changes when making a quantitative analysis.

**Figure 2:**
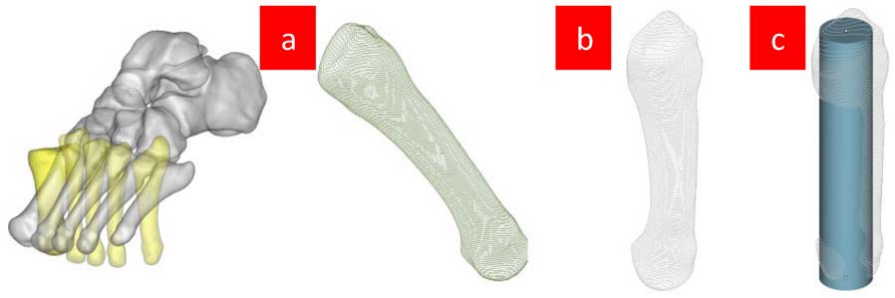
Tomographic reconstruction of the fourth metatarsal after using SCS. Fig. 2a Tomography of the fourth metatarsal before using SCS. Fig. 2b Tomography of the fourth metatarsal after using SCS. Fig. 2c Tomography and sea level of the fourth metatarsal after using SCS. According to the morphological features of the fourth metatarsal, set the average value of the distance between the fourth metartasal's bone tissue to long PAI (the magnitude of principal moments of inertia is minimal) to be the radius. The central axis of the cylinder and the long PAI will superpose. The left figure shows the postures of 1st–5th metartasals before and after using SCS. If a cylinder is placed in Fig. 2a, the bone's tomography is not vertical to the central axis of the cylinder, but a reconstruction of tomography after using SCS is vertical to the central axis of the cylinder (i.e. the principal axis of minimal moment of inertia). The tomography of the non-vertical central axis of the cylinder is determined by the posture of the subject when being measured. A different posture leads to a different tomography. Without using SCS, the development of the tomographic boundary of the bone's surface map is not unique whereas using SCS brings a unique map of the bone's surface.

After using SCS, the bone's surface position is changed. The proposed bone surface mapping is a method to develop the tomographic boundary, suggesting that the tomographic reconstruction of new bone's 3D model. Using Eq. (4), we rebuild the fourth metatarsal of a subject. See Figure 2.

Figure 2 shows that, after being reconstructed, the tomographic image is vertical to the long PAI. The points relative to the sea level on the tomographic image boundary form the altitude.

**Figure 3:**
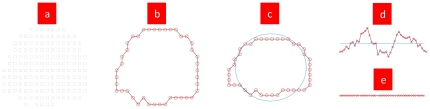
Methods to develop the bone's tomographic image. Fig. 3a Reconstructed tomogram by applying Eq. (4) after using SCS. Fig. 3b Tomographic boundary extracted by Eq. (5). Fig. 3c Circle set up by the average radius of the tomographic image, where the center is on the COM of the tomographic image. Fig. 3d Developed tomographic boundaries by Eqs. (6) and (7) based upon the circumference taken as sea level. Fig. 3e Flattened tomographic boundaries by Eq. (8).

Take one case of superposed tomographic image of the foot bone's COS of the tomography and that of the bone for example. See Figure 3.

Figure 3 shows that Eq. (4) keeps the volume elements to be a square and makes the new tomogram remain closed and continuous after the transformation. The tomographic boundaries constitute the bone's surface, which is drawn from Eq. (5). The tomographic boundary formed by the two PAIs is taken for the prime meridian. Eqs. (6)–(8) are used to develop the bone surface.

**Figure 4:**
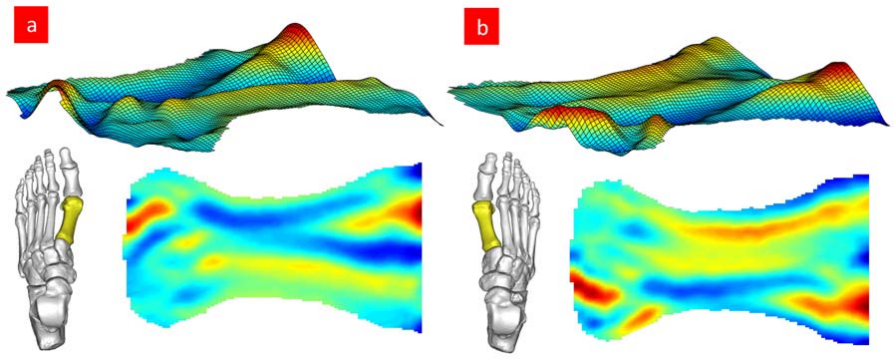
Mapping of human symmetrical bone. Fig. 4a One subject's left foot first metatarsal. Fig. 4b One subject's right foot first metatarsal. The cutting line is the minimal PAI of the first metatarsal. (Some sections of the head and base of the first metatarsal are not on the cutting line. As a result, these sections are not included in the map.)

In human body, the left side bones have symmetrical shapes with the right side ones. To such bones, there is no need to express their surface as a percentage. The mapping of one subject's left/right foot first metatarsal is shown in Figure 4.

**Figure 5:**
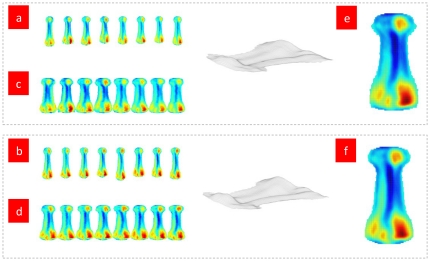
Mapping analysis to the fourth metatarsal. Fig. 5a–5b Mapping of the fourth metatarsal (left foot) of eight wrestlers and volleyball players respectively. Fig. 5c–5d Mapping of the fourth metatarsal (left foot) after standardization of length and width of eight wrestlers and volleyball players, respectively. Fig. 5e–5f Mapping of the fourth metatarsal (left foot) average value from eight wrestlers and volleyball players, respectively. Eq. (3) is applied to SCS of the athletes' fourth metatarsals (left foot) and then the tomography is reconstructed. Eqs. (4)–(7) are employed to develop the reconstructed tomography. See Fig. 5a–5b. To standardize the length and width of the metatarsal map brings the same length and width. See Fig. 5c–5d. Statistical analysis is conducted on the standardized metatarsal map to calculate the average value of the eight metatarsal maps at the same position. See Fig. 5e–5f.

Figures 1, 2, 3, 4 suggest that the mapping method is practical. Whether the method is valuable in bone's biomechanical study and in clinical application will determine the significance of our study. To examine the application of the mapping method in future research, we conduct a mapping analysis to the fourth metatarsal of the eight wrestlers and volleyball players, respectively. See Figure 5.

Figure 5 shows that the standardization of length and width of the map of the positioned development of the fourth metatarsal has brought about the average value map of the fourth metatarsal from many subjects. The result from the comparison of the average value map of the fourth metatarsal from two groups of athletes suggests that the degree of convex and concave surfaces of the fourth metatarsal from the volleyball players is greater than that of the wrestlers, which is consistent with the fact that volleyball players bear greater impact when spiking and blocking.

**Figure 6:**
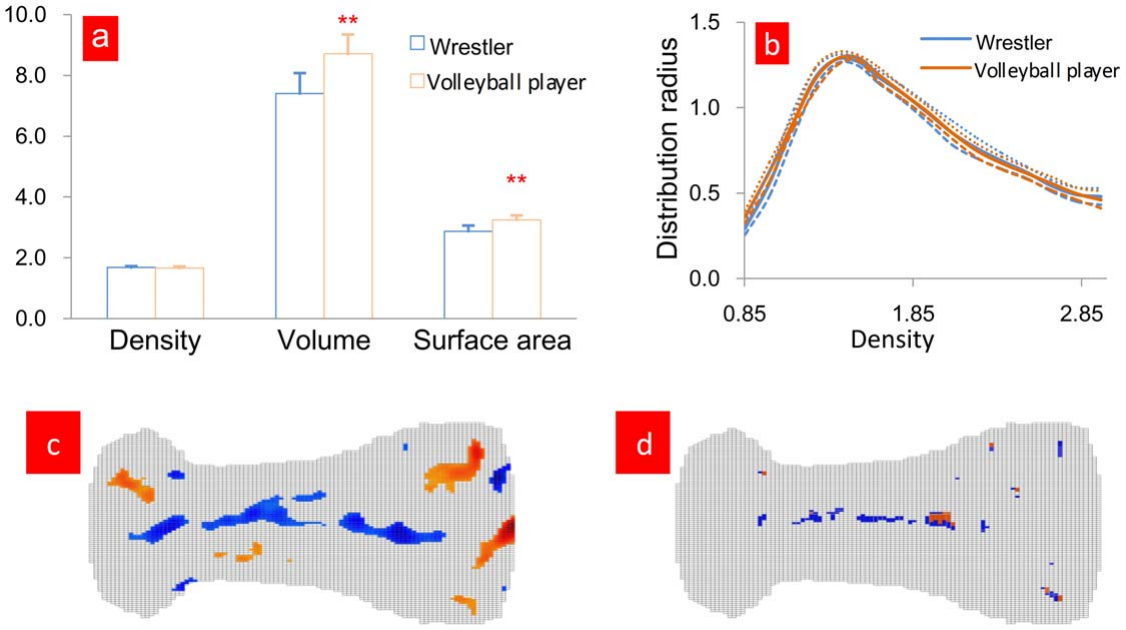
Analysis of bone strength to the fourth metatarsal (left foot) of eight wrestlers and volleyball players, respectively. Fig. 6a Comparison of bone density, volume and area of the fourth metatarsal (left foot) of eight wrestlers and volleyball players. Fig. 6b Relationship between the fourth metatarsal (left foot) bone tissue density and the tissue distribution radius from eight wrestlers and volleyball players. Fig. 6c Differences of the map of the fourth metatarsal (left foot) average value from eight wrestlers and volleyball players. Fig. 6d P value distribution of the fourth metatarsal (left foot) map from eight wrestlers and volleyball players. In Fig. 6a, the bone's density is defined as 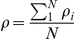, where 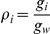, 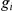 stands for the gray value of the *i*-th volume element, 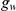 stands for the gray value of water. Volume and area are results from *Mimics* software. In Fig. 6b, for detailed method, see Reference 30. In Fig. 6c, when 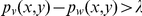, or 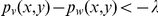, 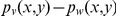 is colored, where 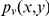 and 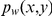 stand for the altitude values for the positions of 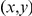 of the fourth metatarsal from volleyball players and wrestlers, respectively, where blue shows 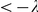 and red 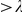 (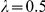). In Fig. 6d, the calculation is done by *SPSS*, where blue shows p<0.05 and red p<0.01.

Bone can function as a body protection and as an aid to body movement [29]. Analysis of bone strength has always been an important issue studied in biomechanics research. The possibility to apply the bone mapping method to analyze bone strength is explored. See Figure 6.

Figure 6 shows the surface features of bone by the mapping method. It also serves as a quantitative method to study the morphological changes of bone, i.e. not only can the indexes of both bone density and the relation between bone tissue density and its distribution radius [30] be used to analyze the bone strength, but also can the mapping method. This can contribute to the bone strength analysis clinically.

### Conclusion

The generalization of Papoulis' theorem [31] elucidates that bone surface shape keeps its geometric invariance, such as rotation, translation or dimension change [32]. This ensures the consistency of the CT scanning results of different positions of bone *in vivo* when its isotropy is ascertained. The uniqueness of the relative consistency of the inertia reference system of principal moments of inertia on direction cosines contributes to the bone surface mapping method. The characters such as the geometric invariance and the uniqueness of the coordinate system of the principal moments of inertia, enable the bone surface mapping method to depict the bone's external morphological characters. This can advance the research of the morphological mechanisms. The experiment of the bone *in vivo* signifies that the bone mapping method adds another research method and supplements the analytical method of the bone's 3D imaging method.

It can be concluded that the asymmetrically shaped and anisotropically distributed bone *in vivo* holds a set of unique PAIs. The body axes randomly set upon the bone's COM, after coordinate transformation, make the body axes coincide with PAIs, indicating that the body axes are the only one set upon PAIs. This applies to the homogeneously asymmetrical geometry as well as the anisotropically asymmetric one. The unique feature of PAIs is the key to determine whether or not the bone *in vivo* can be mapped. This uniqueness has been proved, and thus the mapping method is applicable.

We can draw a map of earth by an artificially defined prime meridian and equator line. But to map more than 200 pieces of human bone, how to employ a physical quantity to define the prime meridian and equator line is what our research team is trying to work out – to use the unique PAIs of the bone to determine the bone's prime meridian and equator line and then to use the mean distance of the bone surface points relative to the bone's COM to set the sea level and finally develop the bone according to the mathematical rules such as reconstruction and boundary detection so that the bone's surface can be mapped.

The morphological research of bone reveals the significance of human evolution. For example, the analysis of the fourth metatarsal of Australopithecus afarensis indicates that the afarensis foot functioned like that of modern humans [33], [34]. Ross and her colleagues' 3D-ID software can allow scientists to use the remains to focus on the shape (instead of size) to identify the sex and ancestry with a better speed and accuracy [35]. These all suggest that there is still much potential to be explored in the morphology of bone. Since human organ, tissue and cell are all asymmetrical geometries like bone which can be mapped accordingly, we believe that the application of this bone surface mapping method will be promising.
